# Evaluation of Dietary Bovine Milk Fat Globule Membrane Supplementation on Growth, Serum Cholesterol and Lipoproteins, and Neurodevelopment in the Young Pig

**DOI:** 10.3389/fped.2019.00417

**Published:** 2019-10-17

**Authors:** Joanne E. Fil, Stephen A. Fleming, Maciej Chichlowski, Gabriele Gross, Brian M. Berg, Ryan N. Dilger

**Affiliations:** ^1^Piglet Nutrition and Cognition Laboratory, University of Illinois, Urbana, IL, United States; ^2^Neuroscience Program, University of Illinois, Urbana, IL, United States; ^3^Mead Johnson Pediatric Nutrition Institute, Evansville, IN, United States; ^4^Division of Nutritional Sciences, University of Illinois, Urbana, IL, United States; ^5^Department of Animal Sciences, University of Illinois, Urbana, IL, United States

**Keywords:** milk fat globule membrane, pig, brain, neurodevelopment, magnetic resonance imaging, novel object recognition, cholesterol, lipoproteins

## Abstract

**Introduction:** Milk fat globule membrane (MFGM) is a protein- and phospholipid-rich membrane that surrounds the lipid droplet in milk. We have previously reported that a diet composed of a combination of prebiotics, bovine MFGM (bMFGM), and lactoferrin (bLf) supported brain development in young pigs. Due to the growing interest of its potential benefits in neurodevelopment, the present study focused on the effects of dietary bMFGM alone using the pig as a translational model.

**Methods:** Male pigs were provided *ad libitum* access to milk replacer with added whey protein-lipid concentrate (source of bMFGM) at 0 (CONT), 2.5 (MFGM-2.5), or 5 (MFGM-5.0) g/L from postnatal day (PND) 2 to 31. Blood was collected from pigs at PND 15 and 31, and pigs underwent behavioral testing using the novel object recognition task starting at PND 25. At PND 31, magnetic resonance imaging was conducted and animals were subsequently euthanized for tissue collection.

**Results:** No group differences in body weight gain or milk intake were observed. At PND 31, few group differences were detected in absolute and relative brain volumes, brain water diffusivity outcomes, or behavioral parameters using the novel object recognition task. Serum lipoprotein was higher in pigs receiving diets with added dietary bMFGM compared with the CONT group. Serum cholesterol and high-density lipoprotein significantly higher (all *P* < 0.05) in the MFGM-2.5 compared with the CONT group. However, cholesterol concentrations within the brain prefrontal cortex and hippocampus did not differ among dietary groups.

**Conclusion:** In this pig model, dietary supplementation with bMFGM was well-tolerated and supported growth and dietary intake similar to the control formula. Added dietary bMFGM was associated with increased serum lipoprotein, but no group differences in early brain cholesterol concentrations, macrostructure, microstructure, or recognition memory pigs at 31 days of age. Further examination of longitudinal brain development and myelination in the pig, particularly at later ages/maturation, is warranted.

## Introduction

Proper early-life nutrition is critical to the growing infant due to direct influence on brain development and effects on growth and cognition later in life. It is well-known that human milk is the gold-standard source of nutrition for understanding the necessary macronutrients and bioactive compounds needed for a developing child (1). However, provision of human milk is not always possible, and in those circumstances, infant formula may be used as an alternative. To support optimal infant development, there is a need to identify biologically-active factors that promote brain development and cognition in the infant. Clinical studies have demonstrated safety and potential benefits of different sources of bovine milk fat globule membrane (bMFGM) or its components in infant formula (2–6), or complementary food for infants and young children (7). We previously observed that a formula with an added prebiotic blend of polydextrose and galactooligosaccharide, bovine milk fat globule membrane (bMFGM), and lactoferrin (bLf) supported neurodevelopment using the neonatal pig model (8). However, effects of the formula with added bMFGM alone on brain development have not yet been studied in developing pigs.

MFGM is a triple-layer membrane that surrounds the lipid droplet in the milk of multiple mammalian species (9–12). It functions to protect the droplet from coalescence and degradation, thereby modulating release of triacylglycerol molecules (13). MFGM had historically been discarded in infant formula and vegetable oil-based ingredients had been used as the main lipid source (14). However, recently interest in MFGM has been growing not only for its functional benefits but also for its potential nutritional significance. Its complex triple layer is composed mainly of phospholipids, sphingolipids, cerebrosides, sterols, proteins, and water (15, 16). Numerous studies have found health benefits associated with various MFGM components, some of which include improved gastrointestinal health and anti-cancer effects (17–20). Likewise, MFGM phospholipids have been shown to support early-life brain development and cognition (21). For example, cognitive scores at 12 months of age were improved in infants receiving formula with MFGM, compared to the control (6). Phosphatidylcholine, phosphatidylethanolamine, and sphingomyelin are just some of the phospholipids within MFGM that may elicit benefits in the structural integrity and signaling functions of cell membranes (22). Moreover, these phospholipids are all a source of choline, an essential nutrient for humans that is vital for proper early life brain development (23). Choline supplementation in rodent models has been shown to improve hippocampal development and spatial memory, while choline deficiency causes greater apoptosis of neurons and glia (23–25). Additionally, dietary sphingomyelin has been shown to support myelination and brain neuroplasticity, two imperative processes in neuronal development (26, 27). An association between sphingomyelin-fortified formula and neurobehavioral development at 18 months in very low birth weight infants has been demonstrated in a pilot, randomized-controlled trial (4). Although individual nutrients constituting MFGM are known to elicit benefits in young animals, research investigating the role of complex MFGM on early-life development is limited.

Therefore, the objective of this study was to assess the influence of added dietary bMFGM on structural brain development, cognitive behaviors, and cholesterol and lipoprotein profiles using the translational pig model. The young pig was used due to its similarities in gastrointestinal system and resemblances in gross neuroanatomy when compared with human infants (28–31). When comparing the total brain volume growth trajectories between humans and pigs, the total brain volume of a 4 month old human infant is similar to the total brain volume of a 4 week old pig (32, 33). We hypothesized that dietary bMFGM would enhance brain structural development and improve performance on the novel object recognition (NOR) behavioral tasks in groups receiving added dietary bMFGM compared with those receiving the control formula.

## Materials and Methods

### Animals and Housing

All animals and experimental procedures were conducted in accordance with the National Research Council Guide for the Care and Use of Laboratory Animals and approved by the University of Illinois at Urbana-Champaign Institutional Animal Care and Use Committee. Fifty-four intact (i.e., not castrated) male pigs were obtained from a commercial swine farm (Carthage Veterinary Services, Carthage, IL) on postnatal day (PND) 2 and transferred to the University of Illinois Piglet Nutrition and Cognition Laboratory where they were artificially reared until PND 30 or 31. The trial was completed in three cohorts with a total of 18 pigs per diet group selected from 16 litters to control for initial body weight and genetics. All pigs were provided with a single dose of prophylactic antibiotic (Excede; Zoetis, Parsippany, NJ) administered at 5.0 mg/kg body weight on day of birth and two doses of *Clostridium perfringens* antitoxin C and D (Colorado Serum Company, Denver, CO; one 5 mL dose given subcutaneously and one 3 mL dose given orally) upon initial arrival to the Piglet Nutrition and Cognition Laboratory on PND 2. Contrary to standard agricultural procedures, pigs on this study were not provided an iron dextran shot as all diets contained adequate concentrations of iron. Additionally, pigs did not receive standard agricultural processing, meaning needle teeth, tails, and testicles were not removed. Pigs were individually housed in custom rearing units, as previously described (8), which allowed pigs to see, hear, and smell neighboring pigs but kept them separate. Lights were automatically turned on and off at 0800 and 2000 h, respectively, with the temperature set at 26.6°C for the first 3 weeks of the study and lowered to 22°C during the last week. Twice daily health observations of individual pigs included visual inspection of pig body condition, demeanor, and fecal consistency.

### Dietary Groups and Feeding Procedures

All milk replacer formulas were produced by Mead Johnson Nutrition (Evansville, IN) using a proprietary blend of nutrients formulated to meet the nutritional needs of growing pigs (34). Individuals involved in conducting and analyzing study results remained blinded to the identity of the dietary treatments until final analyses had been completed. Young pigs (*n* = 18 per group) were provided one of three experimental milk replacer diets with added whey protein-lipid concentrate (source of bMFGM; Lacprodan® MFGM-10, Arla Foods Ingredients) at 0 (CONT), 2.5 (MFGM-2.5), or 5 (MFGM-5.0) g/L (Supplemental Table 1). These concentrations were chosen as they aligned with the fat content of human breast milk and sow milk, which range from 3 to 4% fat and 7 to 8% fat, respectively (35, 36). All three milk replacer powders were reconstituted fresh daily at 200 g of powder per 800 g of water. Using an automated milk replacer delivery system (37), pigs were fed *ad libitum* from 1000 until 0600 the next day (20 h continuous feeding period each day). Individual body weights were recorded daily and health checks recorded twice daily to track the well-being of each pig. Leftover milk from the previous feeding period was subtracted from allotted volume to quantify milk disappearance over the daily feeding period for each pig, which is referred heretofore as milk intake.

### Blood Collection

Blood serum samples were collected on PND 15 as well as at study conclusion on PND 30/31 from 24 pigs (CONT, *n* = 8; MFGM-2.5, *n* = 8; MFGM-5.0, *n* = 8). Blood was collected from the jugular vein into evacuated serum tubes (Becton, Dickenson and Company, Franklin Lakes, NJ), then left at room temperature for at least 45 min to allow for proper clotting. Samples were then centrifuged at 4°C and 1,250 × g for 20 min (Allegra 6R centrifuge, Beckman Coulter Life Sciences, Indianapolis, IN), aliquoted, and stored at −80°C. Serum samples (48 total; 24 mid-study and 24 end-of-study) were analyzed by Dr. Frankie Stentz at the University of Tennessee for analyses of serum cholesterol, triglyceride, high-density lipoprotein (HDL) and low-density lipoprotein (LDL) using validated methods.

### Brain Tissue Collection and Analysis

On PND 30 or 31, all pigs (*n* = 54) were euthanized via exsanguination after CO_2_ asphyxiation. The hippocampus and prefrontal cortex were collected exclusively from the right hemisphere of each pig. Samples were snap-frozen in liquid nitrogen and stored at −80°C. Brain cholesterol was measured in samples (*n* = 36) using a cholesterol colorimetric test (product number MAK043; Sigma Aldrich, St. Louis, MO). The colorimetric test was conducted according to the manufacturer's instructions and all measures were assessed in duplicate. Samples with results above the upper limit of the test's detection range were diluted and re-analyzed.

### Magnetic Resonance Imaging

On PND 30 or 31, pigs (*n* = 36) were transported to the Biomedical Imaging Center at the Beckman Institute for Advanced Science and Technology (University of Illinois, Urbana, IL) and anesthetized upon arrival via an intramuscular injection of a telazol:ketamine:xylazine solution [50.0 mg tiletamine plus 50.0 mg of zolazepam reconstituted with 2.50 mL ketamine (100 g/L) and 2.50 mL xylazine (100 g/L); Fort Dodge Animal Health, Overland Park, KS] at a dose of 0.03 mL/kg of body weight. Once fully sedated, pigs were placed in the MRI scanner and maintained on 2% isoflurane/98% oxygen for the duration of the 60 min scan. Imaging was performed using a Siemens MAGNETOM Prisma 3T scanner with a custom 8-channel head coil specific to the pig. Oxygen saturation levels and heart rate were monitored every 5 min throughout the entirety of the scan. Detailed neuroimaging sequences and post-imaging analysis of volumetric assessment and diffusion tensor imaging have been previously described (38).

### Behavioral Testing

Object recognition memory was tested using novel object recognition (NOR), which has been described in detail previously (37, 39). Testing consisted of habituation, sample, and test phases. During the habituation phase, each pig was placed in an empty testing arena for 10 min each day for 2 days leading up to the sample phase. In the sample phase, the pig was placed in the arena containing two identical objects and given 5 min for exploration. After a delay of ~48 h the pig was returned to the arena for the test phase. During the test phase, the pig was placed in the arena containing one object from the sample phase as well as a novel object and allowed to explore for 5 min. Between trials, objects were removed, immersed in hot water with detergent, and rubbed with a towel to mitigate odor, and the arena was sprayed with water to remove odor cues associated with urine and feces. Objects chosen had a range of characteristics (i.e., color, texture, shape, and size), however the novel and sample objects only differed in shape and size. Only objects previously shown to elicit a null preference were used for testing, and position of the novel object was randomized. Habituation trials began at PND 25 with the test trial concluding on PND 29. The recognition index, the proportion of time spent with the novel object compared to total exploration of both objects, was used to measure recognition memory. Due to a technical error with frame-skipping during some video recordings, some trials were omitted from analysis, however, at least 14 animals per dietary group were included in the final analysis.

### Statistical Analysis

All data were analyzed by an analysis of variance (ANOVA) with a *post-hoc* Tukey adjustment using the MIXED procedure of SAS 9.3 (SAS Inst. Inc., Cary, NC). All statistical models included replicate as a random effect, and fixed main effects of diet and PND, as well as their interaction. The level of significance was set at *P* < 0.05 while trends were defined as 0.05 < *P* < 0.10. Data collected at a single time-point (e.g., MRI outcomes) were analyzed as a one-way ANOVA whereas data collected from the sample animal on more than one occasion (e.g., daily body weights) were analyzed using a repeated-measures ANOVA.

## Results

### Growth Performance and Health

**Figure 1:**
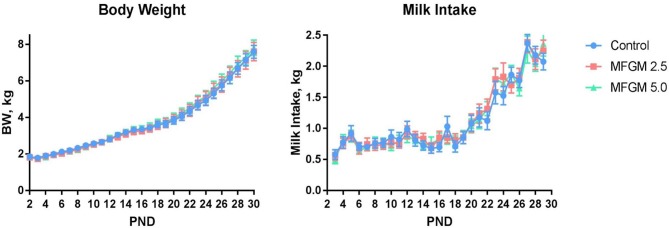
Daily body weights and milk intake of pigs assigned to the control, MFGM-2.5, and MFGM-5.0 dietary treatments over the study duration (18 replicate pigs per treatment). PND 30 is not represented for body weight due to fasting prior to euthanasia. Likewise, milk intake for PND 30 and 31 are not represented, as piglets were fasted the night prior ahead of neuroimaging procedures. MFGM, milk fat globule membrane; PND, postnatal day.

Main effects of diet and PND were observed for daily body weights and milk intake (all *P* < 0.01) as evident in Figure 1. There were no significant effects of diet for average daily body weight gain (ADG), average daily milk intake (ADMI), or the gain-to-feed ratio (G:F) at any interval assessed (all *P* ≥ 0.62; Table 1). Daily health checks showed no difference in pig health status among any dietary group.

**Table 1:** Effect of dietary MFGM supplementation on growth and feeding performance from PND 3-29^a^.

	**Diet**			**Pooled**	
**Measure**	**CONT**	**MFGM-2.5**	**MFGM-5.0**	**SEM**	***P*-value^b^**
ADG, g/day	215	218	225	16	0.89
ADMI, g liquid milk/day	1,100	1,123	1,104	68	0.97
G:F, g BWG: kg liquid milk intake	197	192	204	9	0.62

a *Each mean represents 18 replicate pigs per treatment. CONT, control; MFGM, milk fat globule membrane; SEM, standard error of the mean; PND, postnatal day; BWG, body weight gain; ADG, average daily body weight gain; ADMI, average daily milk intake; G:F, feed efficiency*.

b *P-values derived from mixed model ANOVA*.

### Serum Cholesterol and Lipoprotein Profiles

No interactive effects were observed, so only main effects of diet and PND are reported (Table 2). A main effect of diet was observed between CONT and MFGM-2.5 for serum cholesterol (83.58 vs. 91.82 mg/dl; *P* = 0.012) and HDL (43.48 vs. 46.58 mg/dl; *P* = 0.047). No differences in serum lipoprotein fractions were detected in comparisons of CONT and MFGM-5.0 or MFGM-2.5 and MFGM-5.0. No group differences in the ratio of LDL-to-HDL were detected. A main effect of PND for triglycerides was observed, concentrations of triglycerides concentrations were lower at PND 30 compared to PND 15 (*P* = 0.039; data not shown).

**Table 2:** Concentrations of serum lipoprotein fractions^1^.

	**Diet**			**Pooled**	
**Item^2^**	**CONT**	**MFGM-2.5**	**MFGM-5.0**	**SEM**	***P*-value**
Cholesterol	83.58^b^	97.27^a^	91.82^ab^	4.495	**0.012**
Triglycerides^3^	38.83	50.34	41.97	5.193	0.215
HDL	43.48^b^	50.23^a^	46.58^ab^	3.471	**0.047**
LDL^4^	31.40	36.98	36.86	1.870	**0.049**
LDL:HDL	0.750	0.742	0.794	0.057	0.625

ab *Means without a common superscript letter differ (P < 0.05)*.

1 *Each mean represents 8 replicate pigs per treatment. CONT, control; MFGM, milk fat globule membrane; SEM, standard error of the mean; HDL, high-density lipoprotein; LDL, low-density lipoprotein*.

2 *Units are mg/dL serum*.

3 *A main effect of postnatal day was observed, with triglyceride concentrations decreasing (P < 0.05) over time*.

4 *Mean separation showed no significant difference between groups after Tukey adjustment*.

### Brain Cholesterol

Absolute concentrations of total cholesterol, free cholesterol, and cholesterol ester in the prefrontal cortex and hippocampus did not differ by diet (all *P* ≥ 0.21). Moreover, there were no dietary differences observed in relative concentrations of free cholesterol in the prefrontal cortex or hippocampus (Table 3).

**Table 3:** Concentrations of cholesterol fractions in prefrontal cortex and hippocampus brain region tissues^a^.

	**Diet**			**Pooled**	
**Item**	**CONT**	**MFGM-2.5**	**MFGM-5.0**	**SEM**	***P*-value**
**Absolute^b^**					
**Prefrontal cortex^c^**					
Total cholesterol	35.48	25.91	26.96	6.61	0.417
Free cholesterol	32.40	19.52	22.83	6.37	0.210
Cholesterol ester	3.11	6.41	4.20	2.36	0.611
**Hippocampus**					
Total cholesterol	14.27	14.50	19.17	4.69	0.672
Free cholesterol	7.79	9.55	8.65	1.01	0.484
Cholesterol ester	6.44	4.91	10.49	4.31	0.602
**Relative^d^**					
**Prefrontal cortex**					
Free, % total cholesterol	78.12	88.63	82.97	8.11	0.626
**Hippocampus**					
Free, % total cholesterol	78.17	60.69	68.36	9.43	0.431

a *Each mean represents 12 replicate pigs per treatment. CONT, control; MFGM, milk fat globule membrane; SEM, standard error of the mean*.

b *Units are μg cholesterol fraction/mg brain tissue*.

c *One pig from MFGM-5.0 was excluded from total, free and cholesterol ester analyses due to an abnormally high concentration of cholesterol ester*.

d *Units are expressed as percent relative to total cholesterol content of each sample*.

### Brain Volume Analysis

**Figure 2:**
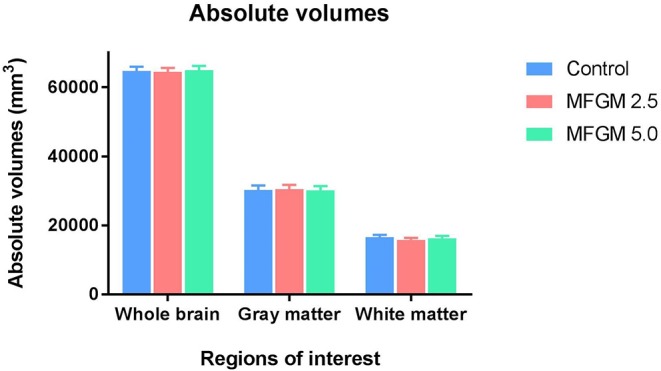
Absolute volumes of whole brain, gray matter, and white matter in each diet (12 replicate pigs per treatment). There were no significant differences in any outcomes due to dietary treatment. MFGM, milk fat globule membrane.

No significant differences between dietary treatments were observed in absolute volumes of whole brain, gray matter, or white matter (Figure 2). Analysis of individual brain regions also showed no dietary differences (all *P* > 0.05; Supplemental Table 2). The relative volume of individual regions relative to total brain volume were also measured for each region of interest (data not shown). Only the relative volume of the cerebral aqueduct was minimally, but significantly, different (*P* = 0.023) across dietary groups.

### Diffusion Tensor Imaging

**Figure 3:**
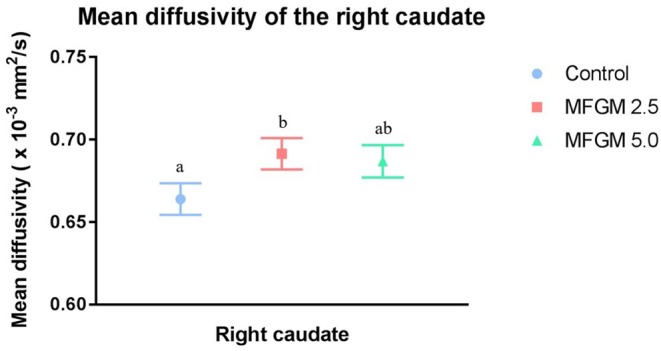
Mean diffusivity values for the right caudate (12 replicate pigs per treatment). Pigs fed MFGM-2.5 exhibited higher (*P* < 0.05) mean diffusivity values compared with CONT pigs. Mean diffusivity in MFGM-5.0-fed pigs did not differ from the other treatments. Means without a common superscript letter differ (*P* < 0.05). MFGM, milk fat globule membrane.

No differences were observed between dietary groups in fractional anisotropy, axial diffusivity, or radial diffusivity measures (Supplemental Table 3). As shown in Figure 3, only mean diffusivity in the right caudate was higher (*P* = 0.044) at 0.69 × 10^−3^ mm^2^/s in pigs provided MFGM-2.5 compared with the mean diffusivity of 0.66 × 10^−3^ mm^2^/s in pigs provided CONT (Supplemental Table 3).

### Novel Object Recognition

**Figure 4:**
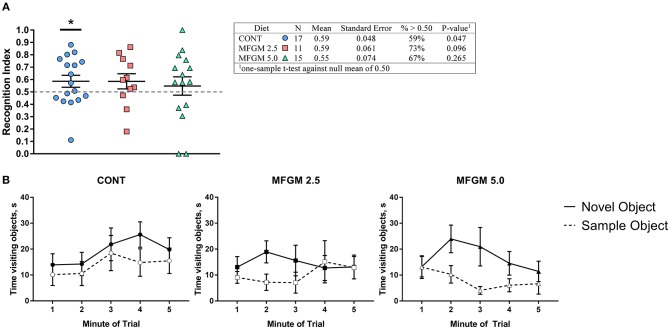
**(A)** Recognition index across the duration of the trial and **(B)** binned by minute. Overall, performance between groups was similar, however only the control group was able to demonstrate a recognition index. Despite having the same average as the CONT group, the MFGM-2.5 group had greater variance, and thus, was not different from a chance value of 0.5. As the plots in **(B)** demonstrate, all groups showed a preference for the novel object at some point during the trial, however both groups fed MFGM demonstrating a null preference at the beginning and end of each trial. CONT, control; MFGM, milk fat globule membrane.

On average, only pigs provided the CONT diet exhibited a recognition index >0.5 (i.e., random chance; *P* < 0.05, Figure 4A). Upon inspection of performance by minute throughout the behavioral trials, all groups demonstrated a preference for the novel object, however, pigs receiving MFGM tended to begin and end the trial with a null preference (Figure 4B). Furthermore, measures of exploratory behavior (mean, frequency, and variance of object visits) were not different between any groups (Supplemental Table 4).

## Discussion

MFGM is a complex membrane that is constructed of many cellular components that are biologically significant in immune protection and gut health (13, 18) and are known to promote neurodevelopment and cognition (3, 13). Bovine MFGM is a primary candidate for routine incorporation in infant formulas with the intent to improve the functional benefits of infant formula for brain development. Previous studies suggest that the inclusion of MFGM or MFGM components in infant formulas was well-tolerated, safe, and may support healthy brain development in infants and young children. To date, research on the influence of dietary bMFGM on brain development and cognition have involved heterogeneous dietary interventions, small sample sizes, or were assessed as a supplement to mammalian milk (6, 8, 40). Because the young pig is considered an appropriate model for a critical neurodevelopmental period (41), we sought to evaluate growth performance, brain structure, cognition, blood and brain cholesterol and lipoprotein concentrations in piglets receiving dietary bMFGM alone. Our results indicate that dietary MFGM was well-tolerated, supported growth and dietary intake similar to the control formula, and influenced serum lipoprotein concentrations. Within the current experimental design, limited group differences were observed in brain macrostructure and microstructure and recognition memory.

### Milk Intake and Growth Performance

Average milk intake did not differ between groups throughout the study. The similarities in milk intake are reflected in average body weights over time, which did not differ between the CONT, MFGM-2.5 and MFGM-5.0 groups. Lack of differences in body weight measures among pigs indicate that MFGM did not influence overall growth. Similar outcomes have been observed in human infants studies, which reported the lipid-rich and/or protein-rich bMFGM sources used in formulas were well-tolerated by infants with no significant differences in body weight gain compared with infants receiving formula with no added bMFGM (2, 6, 21). This suggests that dietary bMFGM does not significantly alter body weight or induce aversive effects on milk intake.

### Serum Lipids

Cholesterol and HDL concentrations increased in serum lipoprotein fractions from pigs in the MFGM-2.5 vs. CONT groups. Increased circulating cholesterol concentrations have also been observed in infants receiving human milk compared with infant formula, attributed to the higher cholesterol content in human milk (42). Serum cholesterol was also higher in infants receiving bMFGM in formula compared with a formula with no added bMFGM (6). Thus, it appears that cholesterol derived from dietary bMFGM may be utilized by the neonatal pig in a manner similar to that of human infants. In rodents, dietary cholesterol has been shown to suppress synthesis of mevalonate and cholesterol by feedback inhibition of 3-hodeozy-3methylglutaryl coenzyme-A reductase activity feed (42); activity of this hepatic enzyme was also in milk-fed compared to formula-fed pigs after 5 days of age (43). The continuous consumption of dietary cholesterol has been suggested to overrule the hepatic control system, which suppress *de novo* synthesis, and results in elevated serum cholesterol (42). In baboons, early-life ingestion of a diet high in cholesterol alters cholesterol metabolism in a manner that provides long-term benefits (44, 45). Likewise, breastfed infants had increased mean total cholesterol and LDL concentrations in infancy, but subsequently displayed lower concentrations in adulthood (46). Interestingly, the MFGM-5.0 pigs did not have higher cholesterol or lipoprotein fraction concentrations than pigs in the CONT and MFGM 2.5 groups. Rosqvist et al. (47) found that the cholesterol and lipoprotein profiles of overweight but healthy men and women were not elevated when given milk fat enclosed by MFGM in contrast to consumption of milk fat without MFGM. They also found changes in gene expression between the two groups. Thus, the lack of a linear response in cholesterol and lipoprotein concentrations with increasing MFGM may be a result of changes in metabolism and/or gene expression in order to maintain homeostasis at the higher MFGM dose. Collectively, our results confirm that cholesterol derived from bMFGM influenced circulating cholesterol concentrations over the course of a 30-d feeding study.

Significantly higher mean serum HDL was observed in the MFGM-2.5 group compared with CONT. HDL is a lipoprotein that functionally regulates cholesterol homeostasis by transporting cholesterol to the liver to be excreted from the body (48, 49). Higher circulating HDL concentrations are typically considered beneficial as this outcome is inversely proportional to the risk of developing cardiovascular disease (49). Increased duration of breastfeeding is known to have a positive linear association with HDL levels in adult life (50), but HDL levels in breastfed and formula fed infants remains debated. Harit et al. (51) observed that serum HDL numerically increased in breastfed infants from 14 weeks to 6 months of age, although this increase was not statistically significant. Additionally, breastfed infants had numerically higher HDL levels at 6 months than the infants provided with a mixed diet of breastfeeding and infant formula. This is contrary to results reported by Timby et al. (6); no differences in HDL concentrations at study enrollment, 4, 6, or 12 months of age were detected between infants receiving formula with added bMFGM, formula without added bMFGM, or a breast-fed reference group. Moreover, HDL levels longitudinally decreased from around 2 months until 12 months of age in all three groups.

In the current study, although addition of dietary bMFGM numerically increased serum lipoprotein fractions, no significant differences in the LDL:HDL ratio were detected. Timby et al. (6) also observed that infants receiving formula with added bMFGM had higher total serum cholesterol and HDL concentrations compared with infants receiving formula without added bMFGM. In addition, no shifts were observed in the LDL:HDL ratio between groups. In simple terms, this may suggest that addition of lipid-rich dietary bMFGM increases serum lipoprotein concentrations in a proportional and regulated manner.

The main effect of PND on serum lipoprotein fractions was also analyzed. Triglycerides were significantly higher on PND 15 compared with PND 30, which is contrary to findings from Srinivasan et al. (52), who observed that serum triglycerides increased in the infant from birth until 6 months of age. However, at 6 months, circulating lipoprotein concentrations began a continual decline through 3 years of age in the human infants. Additionally, data reported by Timby et al. (6) found no longitudinal differences in serum triglycerides of infants receiving infant formulas with or without added bMFGM. However, triglycerides were higher at 4 months of age in the breast-fed reference compared with the group receiving formula without added bMFGM.

### Brain Cholesterol

Absolute and relative concentrations of total cholesterol, free cholesterol, and cholesterol ester in the prefrontal cortex and hippocampus did not differ between diet groups. Similar results were reported for brain lipid composition between mother-reared pups and pups receiving a formula without or with added dietary bMFGM (53). Brain lipids (including phosphatidylethanolamine, phosphatidylserine, and phosphatidylcholine) in the group receiving bMFGM were more similar to the mother-reared group. Free cholesterol concentrations measured from the frontal lobe did not differ between any of the study groups.

Cholesterol in the brain is primarily found in the myelin sheath, which is synthesized locally, mainly by oligodendrocytes and to a lesser extent by neurons and astrocytes (54, 55). Though the blood-brain-barrier may not be fully formed during early development, this barrier prevents direct uptake of circulating cholesterol (56). Our results confirm that increased circulating cholesterol associated with dietary bMFGM ingestion does not appear to influence deposition within the brain. Thus, our null results of cholesterol concentrations within the prefrontal cortex and hippocampus between the three groups are consistent with observations that tightly regulated brain cholesterol concentrations are largely unaffected by diet.

### Magnetic Resonance Imaging

Structural imaging procedures were used to examine the influence of bMFGM ingestion on brain macrostructure and microstructure in the pig. Volumetric analyses of absolute volume of whole brain and anatomic sub-regions revealed no differences due to diet, and only trending significance in the cerebral aqueduct. The cerebral aqueduct had a significantly, but numerically minimal, larger relative brain volume for pigs in the CONT compared with the MFGM-5.0 group; however, it is unclear if this is biologically significant or an artifact of greater relative growth in other areas of the brain.

Tissue microstructure was assessed using diffusion tensor imaging (57). Mean diffusivity measures the average diffusion rate, axial diffusivity is the diffusion rate along the main fiber, radial diffusivity is the diffusion rate perpendicular to the fiber, and fractional anisotropy measures the degree in which diffusion occurs in one direction (58). As myelination increases, it creates a barrier around the neuronal fiber restricting water to diffuse along the neuron rather than perpendicularly. Therefore, as development occurs, one would expect mean diffusivity and radial diffusivity to decrease and axial diffusivity and fractional anisotropy to increase (59). Unexpectedly, we observed greater mean diffusivity in the right caudate of pigs in MFGM-2.5 compared with the CONT group. Additionally, no dose-dependent effects were seen in the right caudate of MFGM-5.0 pigs, which had no significant changes in this brain region. While this may suggest that myelination had not been influenced, we also note that the observed difference was relatively small and may not be biologically significant. Moreover, mean diffusivity is independent of tissue directionality thus does not provide us much insight into brain microstructure without the other diffusion measures. This is contrary to our previous research, which found that addition of bMFGM and other ingredients (prebiotics and bLf) supported structural brain development (8). Chen et al. (60) reported that lactoferrin promotes neurodevelopment through upregulating the brain-derived neurotrophic signaling pathway, suggesting that brain maturation effects observed previously in the pig may have been due to the complementary effects of bMFGM and bLf.

Alternatively, MFGM may support brain development and function through indirect measures that are more evident later in life rather than direct effects shortly after dietary supplementation. It is possible that the pace of brain functional development varies greatly from subject to subject during the first few months of life. As such, MFGM is known to benefit infant gut health and intestinal development by lowering the risk of inflammation and infection (18, 61, 62). Strong evidence suggests that a healthy microbiome during early life may benefit brain development later in life through a bidirectional communication commonly referred to as the microbiota-gut-brain-axis (63–65). Thus, MFGM might partly influence brain development through indirect gut-associated mechanisms, rather than directly altering brain development. Pigs on this study did receive a prophylactic antibiotic on PND 2 due to the greater risk of disease and mortality caused by the transition into a new facility. The use of an antibiotic may have initially decreased the diversity of their microbiome composition which may have metabolically and enzymatically oriented the gut toward a “milk-oriented microbiome” more quickly than had the pigs not received any antibiotic (66). However, single doses of antibiotic administered to pigs shortly after birth have relatively minor effects on gut microbiome development (67). Additionally, all pigs received the antibiotic at the same dosage, thus the results from this study would have not be influenced by this treatment.

It is possible that the effects of adding dietary bMFGM early in life would not be evident until later in the developmental process, meaning that our quantification of brain macrostructure and microstructure occurred too early in the pig. The exact timing of peak myelination in the pig remains unknown, so efforts to align human and pig brain myelination matters is warranted.

### Behavior

In the present experimental design, there were no major group differences in recognition memory-related outcomes. However, examination of performance segmented by minute demonstrates that all pigs in each group demonstrated a preference for the novel object at some point during the trial. Whereas, pigs fed the control diet demonstrated a slight preference for the novel object throughout the entire trial, pigs receiving added dietary bMFGM demonstrated more variability and tended to show a null preference at the beginning and end of the trial and a novelty preference during the middle of the trial. Thus, as a sum of total performance (i.e., Figure 4A), pigs receiving added dietary bMFGM did not show a novelty preference. These data are similar to those previously reported by our lab, wherein pigs fed a diet with added bMFGM, bLf, and a prebiotic blend of polydextrose/galactooligosaccharides did not show memory related improvements on a spatial T-maze task (8). Interestingly, early-life consumption of a diet with polydextrose/galactooligosaccharides increased exploratory behavior and an improved recognition memory in the novel object recognition task in young pigs (37) and was replicated in in a follow-up study (68). These data suggest that prebiotics can greatly influence cognitive outcomes. Thus, the absence of prebiotic supplementation within this study's diet might explain the lack of improvements in memory and exploratory behavior. However, these data do not reflect multiple clinical and preclinical studies supporting an effect of bMFGM on cognition, which may additionally be attributed to the use of various cognitive tasks (21, 40).

In a double-blind randomized controlled trial (DBRCT) in Indonesia, infants received formula with added complex milk lipids (AnmumInfacare; Fonterra Cooperative Group, Aukland, New Zealand) that increased the ganglioside content of the diet from ~ 2–8 weeks to 24 weeks of age. Infants fed formula containing complex milk lipids demonstrated improved hand and eye coordination IQ, performance IQ, and general IQ on the Griffiths Mental Development Scale (3). In a DBRCT in Belgium, 2.5 to 6 year-old children who received chocolate milk with a phospholipid-rich bMFGM concentrate (INPULSE; Büllinger SA, Büllingen, Belgium) for 4 months demonstrated lower parental scoring of internal, external, and total behavioral problems (compared with children who did not receive added bMFGM) (69). Lastly, a DBRCT in Sweden demonstrated infants receiving formula with bMFGM (including an added source of bMFGM; Lacprodan MFGM-10; Arla Foods Ingredients, Viby, Denmark) proteins constituting 4% of the total dietary protein content had higher scores on the Bayley Scales of Infant Development (21).

MFGM has also been shown to improve cognitive development and performance in preclinical rodent models. Rodents fed MFGM exhibited improved performance on spatial and passive avoidance tasks (40), increased hippocampal spine density (70), and increased hippocampal ATP concentrations concurrent with increases in expression of genes related to mitochondrial electron transport (71). In a dose-response study, rats fed diets containing 1% complex milk lipids (Fonterra Research Center, Palmerston North, New Zealand) had increased exploratory behavior in response to a novel object. However, diets containing 0.2% or less did not improve performance on the Morris water maze, novel object recognition, or various operant tasks (72, 73). Collectively, these rodent studies provided the first evidence of a dose-response relationship between MFGM and cognitive performance, so it remains elusive why similar findings have not been corroborated in our pig study. One explanation might be that the influence of MFGM on cognition occurs at a later time-point than what we used in the present study. For instance, Timby et al. (21) provided infants with supplementary MFGM until 6 months of age, but did not measure cognition until 12 months of age. Future studies should look at longitudinal brain development in the pig to see if MFGM supplementation provides beneficial outcomes in development and cognition.

Discrepancies between the behavioral outcomes in this study compared to other studies may be due variations in MFGM composition. Before milks are commercially available, raw milk must undergo various mechanical and thermal processes so that it is safe for human consumption. Thus, MFGM may have an altered structure between varied dairy products (74, 75). Additionally, milk handling procedures such as stirring or cooling and re-heating may influence the membrane's stability (75, 76). In MFGM polar lipid extracts, the lipid packing of the bilayer was changed when cooled from room temperature (26°C) to the temperature range close to a refrigerator (6°C) (76). Thus, different preparation methods may have influenced the biological functions and nutritional properties of MFGM. Moreover, many studies that have found significant influences of MFGM on health and cognition have used individual components of the membrane father than the whole MFGM. Utilizing solely the lipid or protein fraction of MFGM may allow for easier absorption and utilization of the components than if consuming the whole MFGM (77, 78).

## Conclusion

We examined the growth and development of pigs provided supplemental dietary MFGM early in life. The addition of bMFGM to the diet did not appear to negatively impact body weight gain or milk intake, yet increases in serum lipoprotein fractions displayed similar patterns as those found in breastfed infants as well as infants who received added dietary bMFGM. Implications of such findings suggest that infants receiving added bMFGM in formula may exhibit developmental patterns more similar to breastfed infants than to infants given formula without added bMFGM.

To our knowledge, this has been the first study to assess the influence of dietary complex bMFGM on neurodevelopment using neuroimaging outcomes in the pig. Our previous studies have shown that polydextrose, galactooligosaccharides, and bLf in formula in addition to added bMFGM supported neurodevelopment. It is possible that synergistic efficacy among those bioactives influenced the physiological response related to brain development in young pigs. In the present study, direct influence associated with added dietary bMFGM on brain macrostructure and microstructure and recognition memory appeared limited in early life. Longitudinal examination of brain development and myelination in the pig, particularly at later ages, is warranted.

## Data Availability Statement

The datasets generated for this study will not be made publicly available. The data are protected as they were generated as a result of a commercially-funded project.

## Ethics Statement

All animals and experimental procedures were conducted in accordance with the National Research Council Guide for the Care and Use of Laboratory Animals and approved by the University of Illinois at Urbana-Champaign Institutional Animal Care and Use Committee.

## Author Contributions

JF, SF, MC, GG, BB, and RD contributed to the design of the study. JF, SF, and RD interpreted the study and prepared the manuscript. All authors were involved in data acquisition, analysis, interpretation and read and approved the final version of this manuscript.

### Conflict of Interest

The authors declare that this study received funding from Mead Johnson Nutrition. The funder had the following involvement with the study: contribution to study design, reviewing final manuscript before submission.

## Abbreviations

MFGM: milk fat globule membrane
PND: postnatal day
NOR: novel object recognition
CONT: control diet
MFGM-2. 5: experimental diet of 2.5 g MFGM-10/L milk replacer powder
MFGM-5.0: experimental diet of 5.0 g MFGM-10/L milk replacer powder.
