# Supplementary material for: Evaluation of Dietary Bovine Milk Fat Globule Membrane Supplementation on Growth, Serum Cholesterol and Lipoproteins, and Neurodevelopment in the Young Pig
Source: Front Pediatr. 2019 Oct 17;7:417. doi: 10.3389/fped.2019.00417 (PMC6811645; doi:10.3389/fped.2019.00417)
Supplement: Supplementary file 1 [file Table_1.docx]

| **Supplemental Table 1.** Formulated nutrient composition of experimental milk replacers^1^ | | | |
| --- | --- | --- | --- |
|  | **Diet** | | |
| **Nutrient** | **CONT** | **MFGM-2.5** | **MFGM-5.0** |
| Calories, kcal | 1000 | 1001 | 1001 |
| Protein, g | 60 | 60 | 60 |
| Carbohydrate, g | 57 | 55 | 53 |
| Fat, g | 59 | 60 | 62 |
| ARA, mg | 366 | 367 | 368 |
| DHA, mg | 183 | 182 | 183 |
| Calcium, g | 2.16 | 2.21 | 2.20 |
| Phosphorus, g | 1.61 | 1.63 | 1.62 |
| Sodium, g | 1.64 | 1.65 | 1.63 |
| Chloride, g | 0.93 | 0.98 | 0.94 |
| Magnesium, g | 0.24 | 0.24 | 0.23 |
| Potassium, g | 2.11 | 2.06 | 1.98 |
| Copper, mg | 1.59 | 1.59 | 1.59 |
| Iodine, mg | 0.34 | 0.35 | 0.34 |
| Iron, mg | 23.4 | 23.4 | 23.4 |
| Manganese, mg | 2.45 | 2.47 | 2.47 |
| Selenium, mg | 0.07 | 0.07 | 0.07 |
| Zinc, mg | 20.2 | 20.2 | 20.2 |
| Vitamin A, IU | 4022 | 4024 | 4026 |
| Vitamin D, IU | 872 | 872 | 872 |
| Vitamin E, IU | 31 | 32 | 32 |
| Vitamin K, mg | 0.33 | 0.34 | 0.34 |
| Biotin, mg | 0.07 | 0.07 | 0.07 |
| Choline, g | 0.32 | 0.33 | 0.33 |
| Folic Acid, mg | 0.21 | 0.21 | 0.22 |
| Niacin, mg | 10.55 | 10.55 | 10.55 |
| Pantothenic Acid, mg | 9.47 | 9.30 | 9.11 |
| Riboflavin, mg | 3.00 | 2.95 | 2.88 |
| Thiamin, mg | 1.38 | 1.37 | 1.39 |
| Vitamin B6, mg | 1.39 | 1.39 | 1.38 |
| Vitamin B12, µg | 10 | 10 | 10 |
| ^1^Abbreviations: CONT, control; MFGM, milk fat globule membrane. | | | |

| **Supplemental Table 2.** Absolute brain volumes (mm^3^)^1^ | | | | | |
| --- | --- | --- | --- | --- | --- |
|  | **Diet** | | | **Pooled** |  |
| **Region of Interest** | **CONT** | **MFGM 2.5** | **MFGM 5.0** | **SEM** | ***P*-Value** |
| Whole brain | 64689 ± 1228.11 | 64404 ± 1218.23 | 64938 ± 1218.23 | 1228.11 | 0.930 |
| Grey matter | 30237 ± 1318.98 | 30392 ± 1308.41 | 30063 ± 1308.41 | 1318.98 | 0.974 |
| White matter | 16576 ± 677.55 | 15703 ± 672.59 | 16269 ± 672.59 | 677.55 | 0.419 |
| Cerebrospinal fluid | 1804.46 ± 462.52 | 1688.19 ± 458.82 | 2275.62 ± 458.82 | 462.52 | 0.473 |
| Cerebellum | 5958.01 ± 144.83 | 5958.01 ± 144.62 | 5729.25 ± 144.62 | 144.830 | 0.439 |
| Cerebral aqueduct | 16.09 ± 0.54 | 14.96 ± 0.53 | 14.54 ± 0.53 | 0.536 | 0.053 |
| Corpus callosum | 237.59 ± 8.07 | 244.12 ± 8.01 | 244.84 ± 8.01 | 8.066 | 0.598 |
| Fourth ventricle | 19.40 ± 0.86 | 20.00 ± 0.85 | 18.56 ± 0.85 | 0.861 | 0.311 |
| Hypothalamus | 93.22 ± 1.79 | 88.96 ± 1.79 | 90.53 ± 1.79 | 1.787 | 0.249 |
| Lateral ventricle | 334.84 ± 13.94 | 351.33 ± 13.85 | 346.35 ± 13.85 | 13.944 | 0.444 |
| Left caudate | 216.37 ± 9.23 | 229.02 ± 9.16 | 226.60 ± 9.16 | 9.231 | 0.340 |
| Left cortex | 16180 ± 362.96 | 15565 ± 362.96 | 16080 ± 362.96 | 362.960 | 0.447 |
| Left hippocampus | 282.87 ± 6.64 | 285.40 ± 6.59 | 283.96 ± 6.59 | 6.642 | 0.955 |
| Left inferior colliculi | 68.02 ± 1.77 | 66.50 ± 1.77 | 64.93 ± 1.77 | 1.770 | 0.476 |
| Left internal capsule | 520.91 ± 16.17 | 533.64 ± 16.05 | 525.54 ± 16.05 | 16.17 | 0.727 |
| Left olfactory bulb | 1142.68 ± 43.09 | 1109.65 ± 42.77 | 1125.75 ± 42.77 | 43.095 | 0.747 |
| Left putamen-globus pallidus | 123.47 ± 4.08 | 127.57 ± 4.05 | 126.49 ± 4.05 | 4.085 | 0.603 |
| Left superior colliculi | 167.10 ± 2.92 | 160.41 ± 2.92 | 160.08 ± 2.92 | 2.922 | 0.176 |
| Medulla | 1510.90 ± 46.93 | 1483.19 ± 46.55 | 1419.32 ± 46.55 | 46.926 | 0.208 |
| Midbrain | 2131.60 ± 40.86 | 2047.77 ± 40.60 | 2028.54 ± 40.60 | 40.862 | 0.130 |
| Pons | 1285.47 ± 40.66 | 1241.74 ± 40.34 | 1193.84 ± 40.34 | 40.661 | 0.172 |
| Right caudate | 228.33 ± 7.66 | 237.59 ± 7.60 | 235.37 ± 7.60 | 7.662 | 0.477 |
| Right cortex | 16391 ± 448.62 | 15953 ± 447.48 | 15748 ± 447.48 | 448.62 | 0.575 |
| Right hippocampus | 299.55 ± 5.32 | 297.67 ± 5.32 | 298.77 ± 5.32 | 5.321 | 0.969 |
| Right inferior colliculi | 69.11 ± 1.47 | 68.20 ± 1.47 | 67.08 ± 1.47 | 1.467 | 0.621 |
| Right internal capsule | 514.49 ± 10.73 | 521.40 ± 10.64 | 510.94 ± 10.64 | 10.729 | 0.641 |
| Right olfactory bulb | 1136.33 ± 42.72 | 1065.24 ± 42.44 | 1108.01 ± 42.44 | 42.722 | 0.188 |
| Right putamen-globus pallidus | 120.81 ± 2.87 | 121.82 ± 2.85 | 120.36 ± 2.85 | 2.875 | 0.873 |
| Right superior colliculi | 172.15 ± 2.81 | 166.92 ± 2.81 | 166.67 ± 2.81 | 2.814 | 0.312 |
| Thalamus | 1145.38 ± 28.41 | 1138.99 ± 28.22 | 1137.44 ± 28.22 | 28.406 | 0.947 |
| Third ventricle | 20.85 ± 0.81 | 21.05 ± 0.81 | 20.62 ± 0.81 | 0.815 | 0.909 |
| ^1^Data presented are least square means and *P*-values from mixed model ANOVA. | | | | | |

| **Supplemental Table 3.** Diffusion tensor imaging outcomes^1^ | | | | | |
| --- | --- | --- | --- | --- | --- |
|  | **Diet** | | | **Pooled** |  |
| **Region of Interest** | **CONT** | **MFGM 2.5** | **MFGM 5.0** | **SEM** | ***P*-Value** |
| Fractional anisotropy (arbitrary units) |  |  |  |  |  |
| Corpus callosum | 0.36 ± 0.0169 | 0.37 ± 0.0174 | 0.37 ± 0.0174 | 0.017 | 0.844 |
| Cerebellum | 0.24 ± 0.0033 | 0.24 ± 0.0033 | 0.24 ± 0.0036 | 0.004 | 0.944 |
| Left caudate | 0.31 ± 0.0108 | 0.32 ± 0.0108 | 0.34 ± 0.0113 | 0.011 | 0.201 |
| Left hippocampus | 0.34 ± 0.0076 | 0.33 ± 0.0076 | 0.33 ± 0.0079 | 0.008 | 0.633 |
| Left internal capsule | 0.61 ± 0.0072 | 0.60 ± 0.0071 | 0.59 ± 0.0078 | 0.009 | 0.386 |
| Right caudate | 0.30 ± 0.0100 | 0.29 ± 0.0099 | 0.29 ± 0.0103 | 0.010 | 0.812 |
| Right hippocampus | 0.34 ± 0.0082 | 0.34 ± 0.0081 | 0.34 ± 0.0084 | 0.008 | 0.612 |
| Right internal capsule | 0.58 ± 0.0067 | 0.57 ± 0.0067 | 0.57 ± 0.0073 | 0.007 | 0.413 |
| Left side | 0.37 ± 0.0017 | 0.37 ± 0.0017 | 0.37 ± 0.0018 | 0.002 | 0.952 |
| Right side | 0.37 ± 0.0017 | 0.36 ± 0.0017 | 0.37 ± 0.0018 | 0.002 | 0.475 |
| Thalamus | 0.30 ± 0.0036 | 0.30 ± 0.0035 | 0.30 ± 0.0038 | 0.004 | 0.644 |
| T1 white matter | 0.38 ± 0.0017 | 0.38 ± 0.0017 | 0.38 ± 0.0019 | 0.002 | 0.761 |
| Average FA mask | 0.33 ± 0.0015 | 0.33 ± 0.0015 | 0.33 ± 0.0015 | 0.002 | 0.468 |
| Axial diffusivity (AD; × 10^-3^/mm^2^/s ) |  |  |  |  |  |
| Corpus callosum | 0.86 ± 0.0203 | 0.90 ± 0.0207 | 0.88 ± 0.0207 | 0.021 | 0.217 |
| Cerebellum | 0.67 ± 0.0069 | 0.67 ± 0.0069 | 0.68 ± 0.0072 | 0.007 | 0.189 |
| Left caudate | 0.88 ± 0.0158 | 0.89 ± 0.0157 | 0.90 ± 0.0164 | 0.016 | 0.869 |
| Left hippocampus | 0.91 ± 0.0098 | 0.93 ± 0.0098 | 0.93 ± 0.0108 | 0.011 | 0.624 |
| Left internal capsule | 0.85 ± 0.0105 | 0.86 ± 0.0104 | 0.86 ± 0.0106 | 0.011 | 0.856 |
| Right caudate | 0.91 ± 0.0194 | 0.88 ± 0.0191 | 0.91 ± 0.0194 | 0.019 | 0.227 |
| Right hippocampus | 0.92 ± 0.0100 | 0.91 ± 0.0100 | 0.92 ± 0.0104 | 0.010 | 0.883 |
| Right internal capsule | 0.87 ± 0.0125 | 0.87 ± 0.0124 | 0.87 ± 0.0127 | 0.013 | 0.896 |
| Left side | 0.79 ± 0.0073 | 0.79 ± 0.0072 | 0.80 ± 0.0074 | 0.007 | 0.150 |
| Right side | 0.78 ± 0.0076 | 0.79 ± 0.0075 | 0.80 ± 0.0077 | 0.008 | 0.064 |
| Thalamus | 0.78 ± 0.0053 | 0.78 ± 0.0053 | 0.79 ± 0.0055 | 0.006 | 0.160 |
| T1 white matter | 0.79 ± 0.0074 | 0.80 ± 0.0073 | 0.81 ± 0.0075 | 0.008 | 0.109 |
| Average AD mask | 0.76 ± 0.0063 | 0.77 ± 0.0062 | 0.77 ± 0.0068 | 0.007 | 0.740 |
| Radial diffusivity (RD; × 10^-3^/mm^2^/s ) |  |  |  |  |  |
| Corpus callosum | 0.50 ± 0.0117 | 0.51 ± 0.0121 | 0.50 ± 0.0121 | 0.001 | 0.837 |
| Cerebellum | 0.46 ± 0.0053 | 0.46 ± 0.0052 | 0.48 ± 0.0055 | 0.006 | 0.107 |
| Left caudate | 0.54 ± 0.0112 | 0.54 ± 0.0112 | 0.53± 0.0117 | 0.012 | 0.649 |
| Left hippocampus | 0.53 ± 0.0065 | 0.54 ± 0.0064 | 0.53 ± 0.0067 | 0.007 | 0.283 |
| Left internal capsule | 0.29 ± 0.0056 | 0.30 ± 0.0056 | 0.31 ± 0.0062 | 0.006 | 0.196 |
| Right caudate | 0.56 ± 0.0086 | 0.59 ± 0.0086 | 0.58 ± 0.0090 | 0.009 | 0.072 |
| Right hippocampus | 0.53 ± 0.0097 | 0.53 ± 0.0097 | 0.53 ± 0.0099 | 0.010 | 0.846 |
| Right internal capsule | 0.31 ± 0.0055 | 0.32 ± 0.0055 | 0.32 ± 0.0060 | 0.006 | 0.385 |
| Left side | 0.44 ± 0.0039 | 0.44 ± 0.0038 | 0.45 ± 0.0040 | 0.004 | 0.138 |
| Right side | 0.44 ± 0.0037 | 0.44 ± 0.0037 | 0.45 ± 0.0039 | 0.004 | 0.160 |
| Thalamus | 0.50 ± 0.0051 | 0.50 ± 0.0050 | 0.50 ± 0.0052 | 0.005 | 0.440 |
| T1 white matter | 0.44 ± 0.0038 | 0.44 ± 0.0038 | 0.44 ± 0.0040 | 0.004 | 0.209 |
| Average RD mask | 0.46 ± 0.0040 | 0.46 ± 0.0040 | 0.46 ± 0.0043 | 0.004 | 0.672 |
| Mean diffusivity (MD; × 10^-3^/mm^2^/s ) |  |  |  |  |  |
| Corpus callosum | 0.62 ± 0.0105 | 0.64 ± 0.0108 | 0.63 ± 0.0108 | 0.001 | 0.392 |
| Cerebellum | 0.53 ± 0.0056 | 0.54 ± 0.0055 | 0.55 ± 0.0058 | 0.006 | 0.098 |
| Left caudate | 0.66 ± 0.0107 | 0.66 ± 0.0107 | 0.65 ± 0.0111 | 0.011 | 0.912 |
| Left hippocampus | 0.66 ± 0.0062 | 0.67 ± 0.0061 | 0.66 ± 0.0067 | 0.007 | 0.296 |
| Left internal capsule | 0.48 ± 0.0064 | 0.49 ± 0.0064 | 0.50 ± 0.0067 | 0.007 | 0.119 |
| Right caudate | 0.66 ± 0.0096^b^ | 0.69 ± 0.0095^a^ | 0.69 ± 0.0098^ab^ | 0.010 | **0.044** |
| Right hippocampus | 0.66 ± 0.0090 | 0.66 ± 0.0089 | 0.66 ± 0.0092 | 0.009 | 0.867 |
| Right internal capsule | 0.49 ± 0.0063 | 0.50 ± 0.0063 | 0.50 ± 0.0066 | 0.007 | 0.455 |
| Left side | 0.56 ± 0.0049 | 0.56 ± 0.0049 | 0.57 ± 0.0050 | 0.005 | 0.132 |
| Right side | 0.55 ± 0.0048 | 0.56 ± 0.0048 | 0.56 ± 0.0050 | 0.005 | 0.147 |
| Thalamus | 0.59 ± 0.0042 | 0.60 ± 0.0042 | 0.60 ± 0.0044 | 0.004 | 0.311 |
| T1 white matter | 0.56 ± 0.0052 | 0.56 ± 0.0051 | 0.57 ± 0.0053 | 0.005 | 0.089 |
| Average MD mask | 0.56 ± 0.0047 | 0.57 ± 0.0047 | 0.57 ± 0.0051 | 0.005 | 0.701 |
| ^ab^Means without a common superscript letter differ (*P* < 0.05).  ^1^Data presented are least square means and *P*-values from mixed model ANOVA. | | | | | |

| **Supplemental Table 4. Exploratory behavior in the novel object recognition task** | | | | | | | | |
| --- | --- | --- | --- | --- | --- | --- | --- | --- |
|  | **Diet^1^** | | | | | | **Pooled** |  |
| **Measure^2^** | **CONT** | | **MFGM 2.5** | | **MFGM 5.0** | | **SEM** | ***P*-Value^3^** |
| Sample Trial |  |  |  |  |  |  |  |  |
| Total # of object visits | 9.6 ± | 2.32 | 10.4 ± | 2.21 | 12.0 ± | 2.27 | 2.32 | 0.627 |
| Time spent exploring objects, s | 89.0 ± | 28.18 | 80.3 ± | 26.89 | 113.5 ± | 27.50 | 28.18 | 0.535 |
| Mean length of object visits, s/visit | 8.6 ± | 2.54 | 6.2 ± | 2.35 | 10.4 ± | 2.36 | 2.54 | 0.371 |
| Standard deviation of visit length, s | 11.0 ± | 3.06 | 9.3 ± | 3.05 | 12.1 ± | 2.93 | 3.06 | 0.784 |
| Standard error of visit length, s | 3.4 ± | 1.12 | 2.5 ± | 1.12 | 3.9 ± | 1.07 | 1.12 | 0.656 |
| Latency to first object visit, s | 46.8 ± | 15.99 | 51.1 ± | 14.75 | 14.5 ± | 14.80 | 15.99 | 0.137 |
| Test Trial: exploration of all objects |  |  |  |  |  |  |  |  |
| Total # of object visits | 10.7 ± | 1.91 | 11.4 ± | 2.07 | 10.2 ± | 1.91 | 2.07 | 0.883 |
| Time spent exploring objects, s | 114 ± | 15.53 | 94 ± | 17.61 | 95 ± | 15.98 | 17.61 | 0.608 |
| Mean length of object visit, s/visit | 12.7 ± | 1.52 | 10.1 ± | 1.88 | 8.49 ± | 1.61 | 1.88 | 0.166 |
| Standard deviation of visit length, s | 14 ± | 1.61 | 9.94 ± | 2.01 | 9.53 ± | 1.85 | 2.01 | 0.137 |
| Standard error of visit length, s | 4.92 ± | 0.70 | 3.05 ± | 0.87 | 2.69 ± | 0.80 | 0.87 | 0.090 |
| Latency to first object visit, s | 22.1 ± | 13.92 | 17.5 ± | 15.37 | 21.5 ± | 14.00 | 15.37 | 0.956 |
| Test Trial: exploration of the novel object |  |  |  |  |  |  |  |  |
| # of novel object visits | 5.93 ± | 1.25 | 6.21 ± | 1.34 | 5.41 ± | 1.25 | 1.34 | 0.851 |
| Time spent exploring the novel object, s | 69.2 ± | 10.97 | 55.3 ± | 12.44 | 63.1 ± | 11.29 | 12.44 | 0.708 |
| Mean length of novel object visits, s/visit | 13.5 ± | 2.73 | 11.4 ± | 3.08 | 12 ± | 2.90 | 3.08 | 0.814 |
| Standard deviation of novel visit length, s | 11.4 ± | 1.82 | 10.2 ± | 2.09 | 10.1 ± | 2.02 | 2.09 | 0.850 |
| Standard error of novel visit length, s | 4.77 ± | 0.97 | 4.41 ± | 1.09 | 3.74 ± | 1.06 | 1.09 | 0.700 |
| Latency to first novel object visit, s | 43.1 ± | 20.48 | 22.9 ± | 22.84 | 43.9 ± | 21.58 | 22.84 | 0.658 |
| Test Trial: exploration of the sample object |  |  |  |  |  |  |  |  |
| # of sample object visits | 4.67 ± | 0.87 | 5.14 ± | 0.98 | 4.76 ± | 0.89 | 0.98 | 0.931 |
| Time spent exploring the sample object, s | 45 ± | 7.32 | 38.7 ± | 8.30 | 31.9 ± | 7.53 | 8.30 | 0.469 |
| Mean length of sample object visits, s/visit^4^ | 15.4 ± | 2.38^a^ | 9.08 ± | 2.96^a^ | 6.1 ± | 2.62^a^ | 2.96 | **0.038** |
| Standard deviation of sample visit length, s | 14 ± | 3.00 | 8.5 ± | 3.68 | 7.64 ± | 3.36 | 3.68 | 0.323 |
| Standard error of sample visit length, s | 8.2 ± | 2.01 | 3.48 ± | 2.46 | 3.03 ± | 2.24 | 2.46 | 0.178 |
| Latency to first sample object visit, s | 86.2 ± | 18.82 | 31.5 ± | 22.70 | 29.5 ± | 20.28 | 22.70 | 0.068 |
| ^ab^Means without a common superscript letter differ (*P* < 0.05). | | | | | | | | |
| ^1^Data presented are least square means. | | | | | | | | |
| ^2^Abbreviations: #, number; s, second; Δ, change in; min, minute. | | | | | | | | |
| ^3^Data analyzed via one-way ANOVA. | | | | | | | | |
| ^4^Despite diet 2 having a lower SEM (due to a larger sample size), the Brown-Forsythe test for homogeneity of variance assumption revealed diet 2 had a larger variance than diets 1 and 3. Thus, Welch’s ANOVA was used as a non-parametric alternative to a general linear model ANOVA and comparisons were assessed using pairwise two-sided multiple comparisons via the Dwass, Steel, Critchlow-Fligner method. Welch’s ANOVA demonstrated a P-value less than 0.05, however multiple comparison testing revealed no differences between groups. | | | | | | | | |
